# Transcriptome Analysis of Yellow Horn (*Xanthoceras sorbifolia* Bunge): A Potential Oil-Rich Seed Tree for Biodiesel in China

**DOI:** 10.1371/journal.pone.0074441

**Published:** 2013-09-11

**Authors:** Yulin Liu, Zhedong Huang, Yan Ao, Wei Li, Zhixiang Zhang

**Affiliations:** 1 College of Biological Science and Biotechnology, Beijing Forest University, Beijing, China; 2 Laboratory of Systematic Evolution and Biogeography of Woody Plants, College of Nature Conservation, Beijing Forest University, Beijing, China; 3 Academy of Forest, Beijing Forest University, Beijing, China; Cankiri Karatekin University, Turkey

## Abstract

**Background:**

Yellow horn (*Xanthoceras sorbifolia* Bunge) is an oil-rich seed shrub that grows well in cold, barren environments and has great potential for biodiesel production in China. However, the limited genetic data means that little information about the key genes involved in oil biosynthesis is available, which limits further improvement of this species. In this study, we describe sequencing and *de novo* transcriptome assembly to produce the first comprehensive and integrated genomic resource for yellow horn and identify the pathways and key genes related to oil accumulation. In addition, potential molecular markers were identified and compiled.

**Methodology/Principal Findings:**

Total RNA was isolated from 30 plants from two regions, including buds, leaves, flowers and seeds. Equal quantities of RNA from these tissues were pooled to construct a cDNA library for 454 pyrosequencing. A total of 1,147,624 high-quality reads with total and average lengths of 530.6 Mb and 462 bp, respectively, were generated. These reads were assembled into 51,867 unigenes, corresponding to a total of 36.1 Mb with a mean length, N50 and median of 696, 928 and 570 bp, respectively. Of the unigenes, 17,541 (33.82%) were unmatched in any public protein databases. We identified 281 unigenes that may be involved in *de novo* fatty acid (FA) and triacylglycerol (TAG) biosynthesis and metabolism. Furthermore, 6,707 SSRs, 16,925 SNPs and 6,201 InDels with high-confidence were also identified in this study.

**Conclusions:**

This transcriptome represents a new functional genomics resource and a foundation for further studies on the metabolic engineering of yellow horn to increase oil content and modify oil composition. The potential molecular markers identified in this study provide a basis for polymorphism analysis of Xanthoceras, and even Sapindaceae; they will also accelerate the process of breeding new varieties with better agronomic characteristics.

## Introduction

Due to the crises of fossil fuel depletion and worsening global environmental conditions, oil-rich seed plants that can be used to produce renewable and environmentally friendly biodiesel have received much attention [1]–[3]. A variety of vegetable oils that are obtained from rapeseed (canola), soybean, sunflower, peanut, safflower, palm and Jatropha, among others, have been used to produce biodiesel. However, the great majority of these plants are grown on farmland and are used as cooking oil. Under these circumstances, in some developing countries that have limited per capita arable land, the use of food crops to produce biodiesel is not realistic. Therefore, biodiesel production from non-food crops that can be planted in areas that are unsuitable for traditional crops is an ideal solution to this problem [4]–[6].

Yellow horn (*Xanthoceras sorbifolia* Bunge.) is an oil-rich seed shrub that belongs to the Sapindaceae family and has a life span of more than 200 years [7], [8]. The seeds of yellow horn contain abundant oil (55–70%), of which 85–93% is unsaturated fatty acids [9], [10]. According to previous studies, the molecular composition of yellow horn oil is similar to the ideal fatty ester structure for biodiesel [11], [12]. Therefore, it has been identified as a major biodiesel tree species and the Chinese Government provided special support to aid its development because it can produce over 800 gallons of oil per acre of cultivation [13], [14]. Unlike other energy-resource trees, such as palm and Jatropha, that cannot survive low temperatures, it can not only grow well in barren, salty and drought soil, it can also survive temperatures as low as −30 to −41°C. In addition, the yellow horn tree has many other uses, including multiple entries in the Chinese Pharmacopoeia, it can assist in eliminating desertification and erosion, and it is grown as an ornamental tree and used as a source of high-level woody natural oil for cooking [15].

During the last decade many studies have examined yellow horn seed oil; however, these focused on the extraction of oil and methods of biodiesel production from the seed oil [10], [16]–[18]. Unlike other oil crops, such as rapeseed, soybean and Jatropha, no genome-level studies have attempted to determine the oil synthesis metabolic pathway, which could be used to improve the seed yield and oil content. Although conventional breeding strategies continue to play an important role in crop improvement, genetic engineering methods are more rapid and precise, and allow the specific redesigning of crops for target characteristics [19]. For non-model plants, such as yellow horn, for which little or no molecular information is available, next-generation sequencing (NGS) technologies provide a ready means of obtaining genetic information [20]–[22]. The advent of NGS, such as RNA-Seq, in recent years has created unprecedented opportunities for generating genomic information in previously uncharacterised systems. NGS facilitates rapid, inexpensive and comprehensive analyses of complex genomes due to the collection of large-scale sequence data that can be used for gene discovery [23], expression profiling [24], molecular marker development [25] and functional, comparative and evolutionary genomics studies [26]. To date, the transcriptomes of a large number of plants, including many oil crops such as palm [27], peanut [28], sesame [29], safflower [30], rape [31] and Jatropha [32], have been analysed using NGS.

In this study, we report the results of using Roche 454 RNA-seq technology, which can generate sufficiently long sequence reads [33], to analyse the yellow horn transcriptome, which was derived from a pooled sample of DNA from multiple tissue types (buds, leaves, flowers and seeds). The analysis included functional annotation of the transcripts, identification of unigenes that are involved in oil biosynthesis and metabolism, and the discovery of a series of molecular markers (SSRs, SNPs and InDels). These transcripts represent the first yellow horn sequence dataset. We believe the data will open new perspectives for improving and selecting elite yellow horn varieties to produce a greater quantity of high-quality biodiesel.

## Results and Discussion

### Transcriptome Assembly

**Figure 1:**
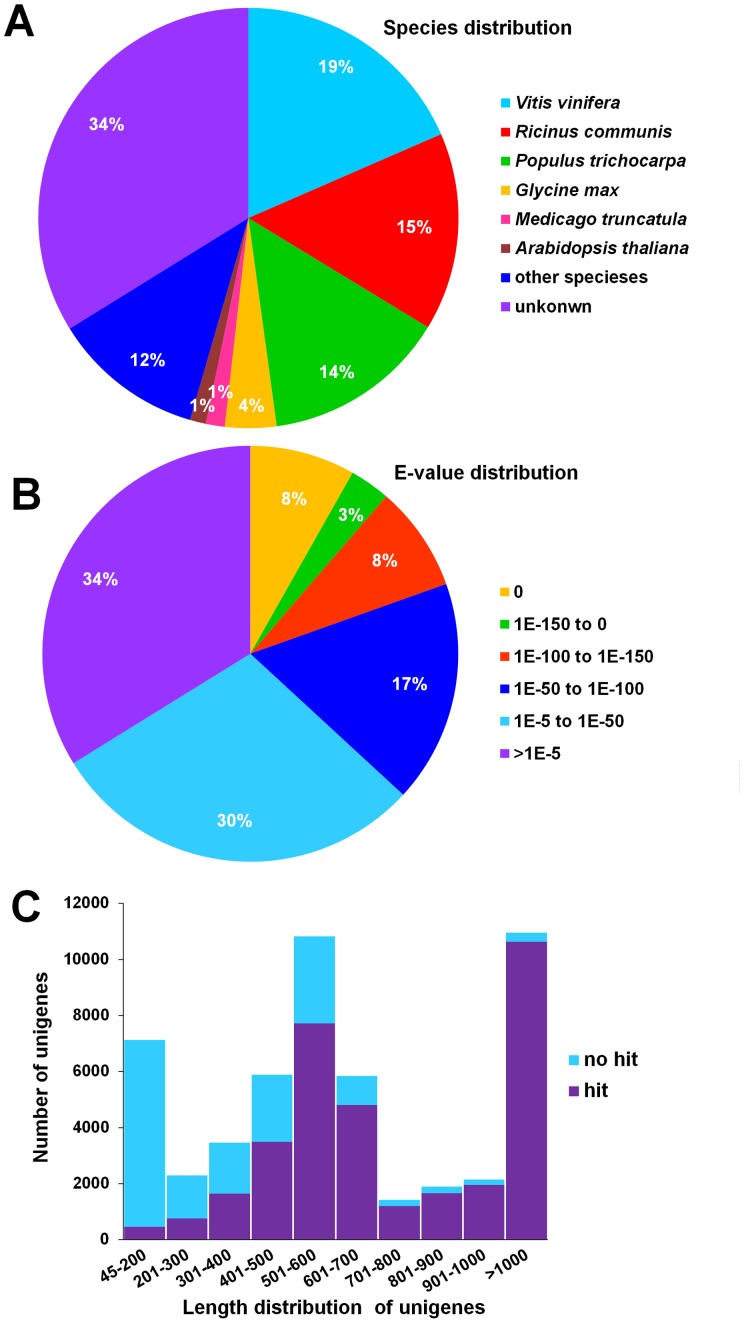
Unigenes functional annotation results. (A) Top-hit species distribution for BLASTx matches for yellow horn unigenes using the following order of priority: NR, TrEMBL and Swiss-Prot. (B) E-value distribution of top BLASTx hits for each unigene. (C) Distribution of unigenes in length with BLASTx hits compared with those without hits.

One and a quarter plates of pyrosequencing reactions were conducted using a 454 GS FLX titanium platform. Approximately 600 Mb of data from 1,221,677 raw reads with a GC content of 43.7% were produced; the read lengths ranged from 23 to 1,478 bp with an average length of 491.1 bp and a median length of 537 bp. After SeqClean was used to cut the adaptors and SMART primers and LUCY2 were used to remove low-quality regions and bases, 88.43% of bases were retained and 1,147,624 trimmed reads (GC content = 43.6%) were generated, with total and average lengths of 530.6 Mb and 462 bp, respectively. Then, 32,165 isotigs (total length = 28,133,950 bp, average length = 874.7 bp, N50 length = 1,116 bp, median length = 732 bp, GC content = 41.40%) and 42,787 singlets (total length = 17,190,382 bp, average length = 401.8 bp, N50 length = 549 bp, median length = 484, GC content = 42.70%) were assembled using Newbler2.6. Finally, after a last assembly using CD-HIT for isotigs and singlets, a dataset of 51,867 unigenes (45 to 10,088 bp with a GC content of 41.37%) was obtained, corresponding to 36.1 Mb with mean, N50 and median lengths of 696, 928 and 570 bp, respectively. Of the unigenes 7,127 (13.74%) were equal to or shorter than 200 bp and 10,960 (21.13%) were longer than 1,000 bp (Table 1, Figure 1C).

**Table 1:** Summary of yellow horn 454 sequencing and assembly.

Items	Raw reads	Trimmed Reads	Isotigs	Singlets	Unigenes
Total number	1,221,677	1,147,624	32,165	42,787	51,867
Total bases (bp)	599,995,187	530,556,412	28,133,950	17,190,382	36,093,173
Average length (bp)	491.1	462.3	874.7	401.8	695.9
N50 (bp)	–	–	1,116	549	928
Median (bp)	537	511	732	484	570
Range of length (bp)	23–1,478	45–965	45–10,088	45–823	45–10,088
GC content (%)	43.70%	43.64%	41.40%	42.70%	41.37%

### Characterisation of Non-redundant Unigenes

To understand their functions, the 51,867 yellow horn unigenes were annotated using BLASTx alignment with an E-value cut-off of 10^−5^ against the following protein databases: NR, Swiss-Prot, CDD, Pfam, TrEMBL, COG, GO, KEGG and TAIR. A total of 33,924 (65.41%), 33,872 (65.31%), 30,504 (58.81%), 28,460 (54.87%), 26,643 (51.36%), 24,412 (47.07%), 24,258 (46.77%), 11,181 (21.56%) and 7,415 (14.30%) unigenes had significant matches with sequences in the NR, TrEMBL, Pfam, TAIR, GO, Swiss-Prot, CDD, COG and KEGG databases, respectively (Table 2). Of the unigenes, 34,326 (66.18%) were described in at least one database with high homology (Figure 1A) with unigenes from *Vitis vinifera* (9,580, 18.47%), *Ricinus communis* (7,900, 15.23%), *Populus trichocarpa* (7,337, 14.15%), *Glycine max* (2,052, 3.96%), *Medicago truncatula* (755, 1.46%), *Arabidopsis thaliana* (635, 1.22%), and other species (6,067, 11.70%) (Figure 1B). However, the remaining 17,541 (33.82%) were unmatched (70.55% of unigenes <500 bp and 1.90% of unigenes >1,000 bp) (Figure 1C), suggesting that longer sequences were more likely to have BLAST hits and shorter sequences may have been either too short to get hits or lacked a characterised protein domain, which resulted in false-negative results. Because public databases contain little genomic and transcriptomic information for yellow horn, these unmatched unigenes may represent putative tissue-specific novel genes or non-coding regions.

**Table 2:** Functional annotation of yellow horn unigenes in public protein databases.

Database	Number of annotated unigenes	Percentage (%)
**NR**	33,924	65.41%
**TrEMBL**	33,872	65.31%
**Pfam**	30,504	58.81%
**TAIR**	28,460	54.87%
**GO**	26,643	51.36%
**Swiss-Prot**	24,412	47.07%
**CDD**	24,258	46.77%
**COG**	11,181	21.56%
**KEGG**	7,415	14.30%
**Total**	34,326	66.18%

### Functional Classification of Unigenes by COG, GO and KEGG

To further evaluate the completeness of our transcriptome library and the effectiveness of our annotation process, we used the annotated unigene sequences to search for genes involved in COG classifications, GO assignments and KEGG pathway assignments to predict and classify their functions.

**Figure 2:**
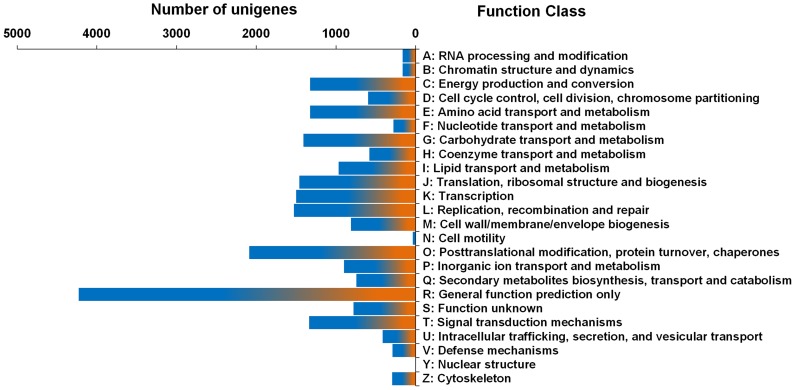
Clusters of orthologous groups (COG) classifications of yellow horn unigenes.

Overall, 11,181 unigenes were assigned into 24 COG function categories. Among them, the cluster for “general function prediction only” represented the largest group (4,227, 37.81%), followed by “posttranslational modification, protein turnover, chaperones” (2,086, 18.66%), “replication, recombination repair” (1,526, 13.65%), “transcription” (1,497, 13.39%) and “translation, ribosomal structure and biogenesis” (1,459, 13.05%). However, few unigenes were assigned into “cell motility” (36, 0.32%) and “nuclear structure” (3, 0.03%). Additionally, 967 (8.65%) unigenes were assigned into the category “lipid transport and metabolism” (Figure 2).

**Figure 3:**
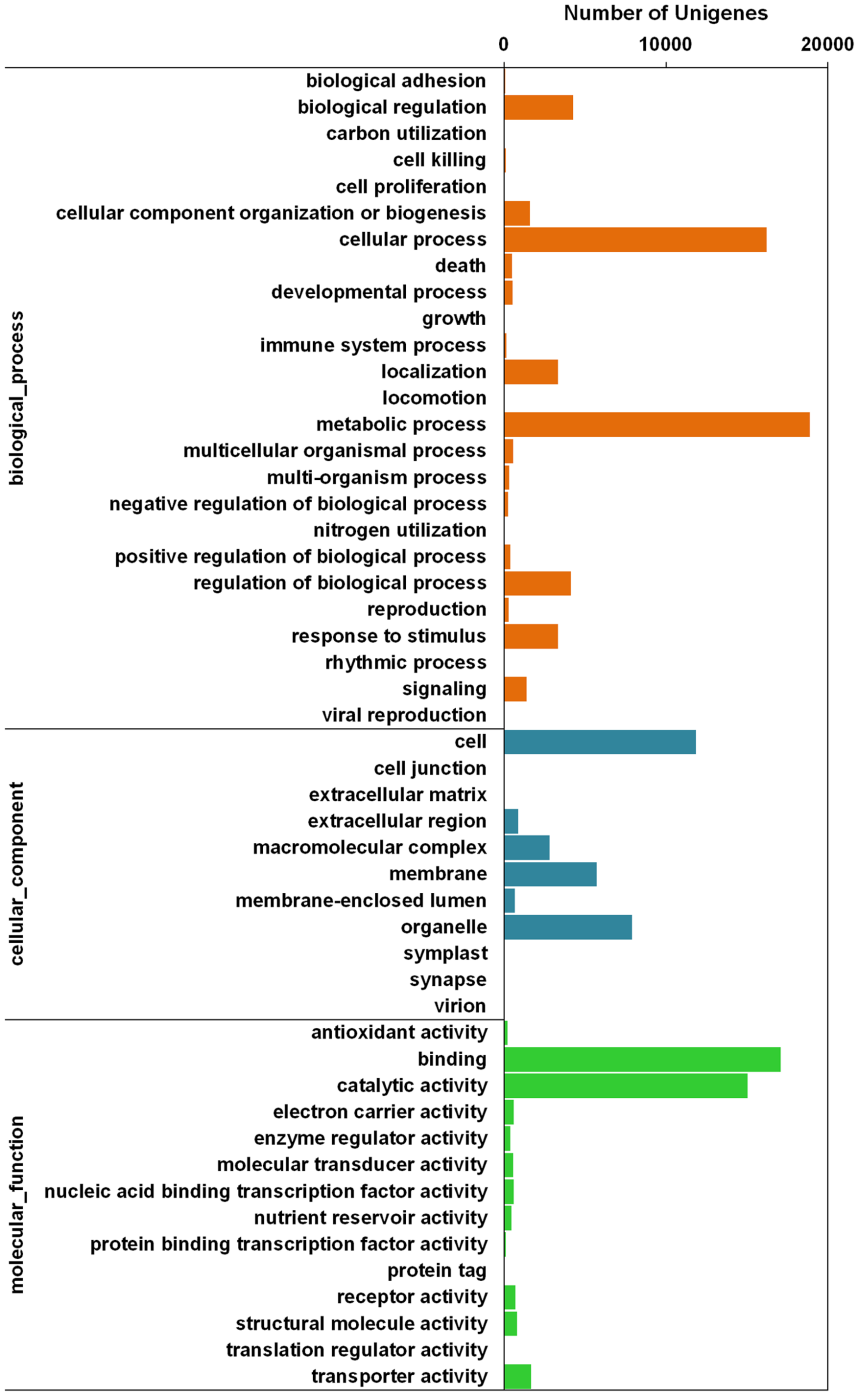
Gene Ontology (GO) categories assigined to the yellow horn unigenes.

Of the unigenes, 26,643 were assigned into the three main GO functional categories and then into 50 sub-categories (Figure 3). For the three main categories, 23,978 unigenes (90.03%) were assigned into the largest category, molecular function, followed by biological process (22,187, 83.28%) and cellular component (15,012, 56.35%). The biological process category was assigned into 25 sub-categories; the most abundant was “metabolic process”, which contained 18,891 unigenes (36.42% of the total), indicating that these genes were enriched in the yellow horn transcriptome libraries. The cellular components category was divided into 11 small groups; the largest sub-category was “cell”, which included 11,855 (22.86% of the total) unigenes. For molecular function, 23,978 unigenes were categorised into 14 GO terms; the majority fell into “binding” and “catalytic activity”, which contained 17,084 and 15,045 unigenes, respectively.

In addition, a total of 7,415 unigenes were assigned to 182 pathways through KEGG pathway assignment. Among them, the largest represented category was “metabolism”, containing 3,862 unigenes, followed by “genetic information processing” and “cellular processes”, which contained 2,736 and 1,070 unigenes, respectively. The pathway “ribosome” involved the largest number of unigenes (475), but both the “primary bile acid biosynthesis” and “nitrotoluene degradation” pathways contained only one unigene. In addition, 548 unigenes were mapped to 15 pathways in the sub-category “lipid metabolism” (Table S1).

### Unigenes Related to FA Biosynthesis

In oil plants, fatty acids are stored as a form of TAG and their biosynthesis pathway can be divided into three steps [34]. The first step is *de novo* biosynthesis of fatty acids; this process occurs in plastids and is catalysed mainly by the fatty acid synthase complex (FAS). The second step is the synthesis of triacylglycerol (TAG), which occurs in the endoplasmic reticulum (ER), and the last is the formation of oil bodies (OBs), where TAG is combined with oleosin to form an oil body, which is released from the ER into the cytoplasm [35], [36].

**Figure 4:**
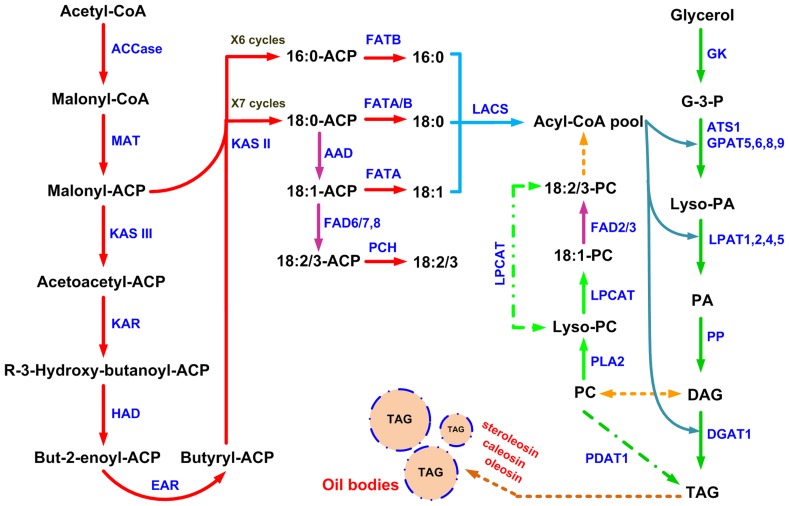
Overview of de novo fatty acid (FA) and triacylglycerol(TAG) biosynthesis pathways. Indentified enzymes include: ACCase, α-acetyl-CoA carboxylase carboxyl transferase (EC:6.4.1.2); MAT, Malonyl-CoA-ACP transacylase (EC:2.3.1.39); KAS, 3-Ketoacyl ACP synthase (KASII, EC: 2.3.1.179; KAS III, EC: 2.3.1.180); KAR, 3-Ketoacyl ACP reductase (EC:1.1.1.100); HAD, 3R-hydroxymyristoyl ACP dehydrase (EC:4.2.1.-); EAR, enoyl-ACP reductase I (EC:1.3.1.9); FATA/B, fatty acyl-ACP thioesterase A/B (EC:3.1.2.14 3.1.2.-); AAD, acyl-ACP desaturase (EC:1.14.19.2); PCH, palmitoyl-CoA hydrolase (EC:3.1.2.2); ACSL, long-chain acyl-CoA synthetase (EC:6.2.1.3); FAD2/6, Δ12(ω6)-Desaturase (EC:1.14.19.-); FAD3/7/8, Δ15(ω3)-Desaturase (EC:1.14.19.-); GK, glycerol kinase (EC:2.7.1.30); ATS1/GPAT, glycerol-3-phosphate acyltransferase (EC:2.3.1.15); LPAT, lysophosphatidyl acyltransferase (EC:2.3.1.51); PP, phosphatidate phosphatase (EC:3.1.3.4); DGAT1, diacylglycerol *O*-acyltransferase 1 (EC:2.3.1.20); PDAT1, phospholipid: diacylglycerol acyltransferase 1 (EC:2.3.1.158); LPCAT, lysophosphatidylcholine acyltransferase (EC:2.3.1.23 2.3.1.67); PLA2, Phospholipase A2 (EC:3.1.1.4). Lipid substrates are abbreviated: 16∶0, palmitic acid; 18∶0, stearic acid; 18∶1, oleic acid; 18∶2, linoleic acid.

According to the KEGG pathway assignment and functional annotation of the unigenes, 40 unigenes were annotated as encoding ten key enzymes involved in FAs biosynthesis (Table 3). The reconstructed pathway of FAs biosynthesis was based on these identified enzymes (Figure 4). First, acetyl-CoA carboxylase (ACCase, EC: 6.4.1.2), as a rate-limiting enzyme in the FAs biosynthesis pathway, catalyses acetyl-CoA to form malonyl-CoA [37]; 16 unigenes that encode its four subunits were identified (four for α-carboxyltransferase, three for β-carboxyltransferase, six for biotin carboxylase and three for biotin carboxyl carrier protein). Next, a series of condensation reactions of malonyl-CoA with a growing ACP-bound acyl chain are catalysed by FAS, consecutively adding two carbon units per cycle over six or seven cycles to form 16∶0-ACP or 18∶0-ACP, which can then be catalysed by acyl-ACP desaturase (AAD, EC: 1.14.19.2) to form 16∶0-ACP or 18∶1-ACP [38]. Nineteen unigenes that encoded the five components of FAS were found, including one that encoded malonyl-CoA-ACP transacylase (MAT, EC: 2.3.1.39), eight that encoded 3-ketoacyl ACP synthase (KAS; seven for KAS II (EC: 2.3.1.179) and one for KAS III (EC: 2.3.1.180)), six that encoded 3-ketoacyl ACP reductase (KAR, EC: 1.1.1.100), two that encoded 3-hydroxymyristoyl ACP dehydrase (HAD, EC: 4.2.1.-) and two that encoded enoyl-ACP reductase (EAR, EC:1.3.1.9). Moreover, nine unigenes coded the acyl carrier protein (ACP), an essential cofactor of FAS. After this, under the control of acyl-ACP thioesterase (FAT, EC: 3.1.2.14 3.1.2.-) and palmitoyl-CoA hydrolase (PCH, EC: 3.1.2.2), free FAs are released from the acyl carrier protein (ACP). Two unigenes that encoded FATA, three that encoded FATB and one that encoded PCH which is also involved in FA elongation were identified in this study.

**Table 3:** Enzymes/protein related to FA biosynthesis and metabolism identified by annotation of the yellow horn unigenes.

Symbol	Enzymes/Protein	EC Number	Number of unigenes
**Fatty acid biosynthesis**			
accA	α-acetyl-CoA carboxylase carboxyl transferase	EC:6.4.1.2	4
accB, bccP	acetyl-CoA carboxylase biotin carboxyl carrier protein	–	3
accC	acetyl-CoA carboxylase/biotin carboxylase	EC:6.4.1.2	6
accD	β-acetyl-CoA carboxylase carboxyl transferase	EC:6.4.1.2	3
ACP	acyl-carrier protein		9
MAT	Malonyl-CoA-ACP transacylase	EC:2.3.1.39	1
KASII	3-Ketoacyl ACP synthase II,	EC:2.3.1.179	7
KAR	3-Ketoacyl ACP reductase	EC:1.1.1.100	6
KASIII	3-Ketoacyl ACP synthase III	EC:2.3.1.180	1
EAR	enoyl-ACP reductase I	EC:1.3.1.9	2
HAD	3R-hydroxymyristoyl ACP dehydrase	EC:4.2.1.-	2
FATA	fatty acyl-ACP thioesterase A	EC:3.1.2.14 3.1.2.-	2
FATB	fatty acyl-ACP thioesterase B	EC:3.1.2.14 3.1.2.-	3
**Fatty acid elongation**			
KCS	3-ketoacyl-CoA synthase	EC:2.3.1.-	20
KR	β-keto reductase	EC:1.1.1.-	3
PHS1	3-hydroxy acyl-CoA dehydratase	EC:4.2.1.-	6
MECR	mitochondrial trans-2-enoyl-CoA reductase	EC:1.3.1.38	1
TER	enoyl reductase	EC:1.3.1.-	7
PCH	palmitoyl-CoA hydrolase	EC:3.1.2.2	1
PPT	palmitoyl-protein thioesterase	EC:3.1.2.22	3
**Fatty acid desaturation**			
AAD	acyl-ACP desaturase	EC:1.14.19.2	6
FAD2	Δ12(ω6)-Desaturase (endoplasmic reticulum)	EC:1.14.19.-	5
FAD3	Δ15(ω3)-Desaturase (microsomal)	EC:1.14.19.-	2
FAD6	Δ12(ω6)-Desaturase (chloroplast)	EC:1.14.19.-	1
FAD7	Δ15(ω3)-Desaturase (chloroplast)	EC:1.14.19.-	5
FAD8	Δ15(ω3)-Desaturase (chloroplast)	EC:1.14.19.-	1
**Fatty acid metabolism**			
ACAA1	acetyl-CoA acyltransferase 1	EC:2.3.1.16	8
atoB	acetyl-CoA C-acetyltransferase	EC:2.3.1.9	6
ACADM	acyl-CoA dehydrogenase	EC:1.3.99.3	7
ACOX	acyl-CoA oxidase	EC:1.3.3.6	11
ACSL	long-chain acyl-CoA synthetase	EC:6.2.1.3	16
ADH	alcohol dehydrogenase	EC:1.1.1.1	15
ALDH	aldehyde dehydrogenase (NAD+)	EC:1.2.1.3	16
MFP2	enoyl-CoA hydratase/3-hydroxyacyl-CoA dehydrogenase	EC:4.2.1.17 1.1.1.35 1.1.1.211	4

In addition, 16 unigenes that encode long-chain acyl-CoA synthetases (LACS), which catalyse the esterification of free FAs to CoA upon arrival in the cytoplasm [39], and seven that encoded acyl CoA binding protein (ACBP), which binds medium- and long-chain acyl-CoA esters with very high affinity and may function as an intracellular carrier of acyl-CoA esters [40], were also identified based on the functional annotation of the transcriptome.

As has been reported previously, overexpression of ACCase, a crucial enzyme in fatty acid synthesis, can alter the fatty acid composition of seeds and increase the fatty acid content, which would lead to an increased oleic acid content and seed yield [41], [42]. Most research on FAS has concentrated on ACP and KAS. Functional expression of an ACP from *Azospirillum brasilense* in *Brassica juncea* can improve the content of 18∶1 and 18∶2 in seeds, enhance the ratio of monounsaturated (18∶1) to saturated fatty acids, increase the ratio of 18∶2 to 18∶3 and reduce the erucic acid content (22∶1) [43]. In plastids, three types of KAS are found: KAS I, KAS II and KAS III. KAS II catalyses 16∶0-ACP to elongate to 18∶0-ACP and KAS III condenses acetyl-CoA with malonyl-ACP to form 4∶0-ACP. Using hairpin RNAi to reduce the activity of KAS II can lead to an increase in 16∶0 accumulation, up to about 53% of the total, but some transgenic offspring are deformed during early embryonic development [44]. Overexpression of KAS III can also improve 16∶0 accumulation, but the rate of lipid synthesis is reduced [45]. In our study, we did not find any unigenes that encoded KAS I, which is highly active with acyl-ACP with chain lengths from C2 to C14, is far less effective for 16∶0-ACP and almost inactive for 18∶0-ACP [46].

### Unigenes Related to TAG and OBs Biosynthesis

In the suggested pathway for TAG biosynthesis [47], [48], a total of 33 unigenes that encode six enzymes were found. As the data in Table 4 and Figure 4 show, initially, one unigene that encodes glycerol kinase (GK, EC: 2.7.1.30), which catalyses glycerol to form glycerol-3-phosphate (G-3-P), an initial substrate in the Kennedy pathway, was detected. Then, 12 unigenes that encode the key component of TAG biosynthesis, glycerol-3-phosphate acyltransferase (ATS1 and GPAT, EC: 2.3.1.15) (three for ATS1, one for GPAT5, three for GPAT6, two for GPAT8 and three for GPAT9), which catalyses the first step of the Kennedy pathway, and 11 unigenes that encode lysophosphatidyl acyltransferase (LPAT, EC: 2.3.1.51) were identified (four for LPAT1, two for LPAT2, three for LPAT4 and two for LPAT5). Under catalysis by these two enzymes, sequential esterification of acyl chains from acyl-CoA to the positions of *sn-1* and *sn-2* of G-3-P occur to form lysophosphatidic acid (Lyso-PA) and phosphatidic acid (PA), respectively. The next reaction is catalysed by phosphatidate phosphatase (PP, EC: 3.1.3.4), a key regulator of lipid homeostasis, which was encoded by four unigenes; it plays a role in the removal of the phosphate group from PA and forms diacylglycerol (DAG), which is an essential intermediate in the biosynthesis of phosphatidylcholine (PC). Finally, two enzymes, diacylglycerol *O*-acyltransferase (DGAT, EC: 2.3.1.20) and phospholipid: diacylglycerol acyltransferase (PDAT, EC: 2.3.1.158), which use acyl-CoA and phospholipids as acyl-donors, respectively, transfer an acyl group to the *sn-3* position of DAG to produce TAG. Only one unigene that encoded DGAT1 and four that encoded PDAT1 were identified in the yellow horn transcriptome. In addition, two enzymes, phospholipase A2 (PLA2, EC:3.1.1.4) and lysophosphatidylcholine acyltransferase (LPCAT, EC:2.3.1.23 2.3.1.67) which may regulate the acyl editing were also identified (Table 4 and Figure 4).

**Table 4:** Enzymes related to TAG biosynthesis and metabolism identified by annotation of the yellow horn unigenes.

Symbol	Enzymes/Protein	EC Number	Number of unigenes
**TAG biosynthesis**			
GK	glycerol kinase	EC:2.7.1.30	1
ATS1	glycerol-3-phosphate acyltransferase (chloroplast)	EC:2.3.1.15	3
GPAT5	glycerol-3-phosphate acyltransferase 5	EC:2.3.1.15	1
GPAT6	glycerol-3-phosphate acyltransferase 6	EC:2.3.1.15	3
GPAT8	glycerol-3-phosphate acyltransferase 8	EC:2.3.1.15	2
GPAT9	glycerol-3-phosphate acyltransferase 9	EC:2.3.1.15	3
LPAT 1	lysophosphatidyl acyltransferase 1 (chloroplast)	EC:2.3.1.51	4
LPAT 2	lysophosphatidyl acyltransferase 2	EC:2.3.1.51	2
LPAT 4	lysophosphatidyl acyltransferase 4	EC:2.3.1.51	3
LPAT 5	lysophosphatidyl acyltransferase 5	EC:2.3.1.51	2
PP	phosphatidate phosphatase	EC:3.1.3.4	4
DGAT1	diacylglycerol *O*-acyltransferase 1	EC:2.3.1.20	1
PDAT1	phospholipid:diacylglycerol acyltransferase 1	EC:2.3.1.158	4
ACBP	acyl CoA binding protein		7
**Acyl editing**			
PLA2	Phospholipase A2	EC:3.1.1.4	1
LPCAT	lysophosphatidylcholine acyltransferase	EC:2.3.1.23 2.3.1.67	5
**TAG metabolism**			
TGL4	TAG lipase	EC:3.1.1.3	3

Overexpression of a plastidial safflower GPAT and an *Escherichia coli* GPAT in *Arabidopsis* can improve the seed oil content, with average increases of 22 and 15%, respectively [49]. Similarly, substantial increases of 8 to 48% in seed oil content and increases in both overall proportions and amounts of very-long-chain fatty acids in seed TAGs were obtained by overexpression of a mutant form of yeast LPAT in *Arabidopsis* and *Brassica napus* [50]. DGAT is a key enzyme regulating the rate of the Kennedy pathway. It has four types, and we detected DGAT1 in this study. Ectopic expression of DGAT will improve the oil content in seeds, which has been confirmed in *Arabidopsis*, soybean and maize [51]–[53]. In addition, compared to the single unigene that encoded GPAT1, the finding of four unigenes that encoded PDAT1 in our study provides further evidence that yellow horn has the potential to channel fatty acids that are incorporated in membrane lipids, such as PC, into TAG biosynthesis.

After biosynthesis, pools of TAGs can be stored as a form of OB surrounded by a membrane composed of a layer of phospholipids embedded with several proteins: oleosin, caleosin and steroleosin in mature seeds [35], [54]. According to the annotations of the unigenes, nine encoded oleosin, seven encoded caleosin and four encoded steroleosin (Table S2). Oleosin is the most abundant structural protein in OBs; it helps stabilise OBs through increased space bit resistance and charge repulsion, preventing fusion of OBs [35], [55]. Caleosin is not only involved in the synthesis and metabolism of OBs, but may also be associated with plant drought tolerance [55], [56]. Steroleosin-like proteins may represent a class of dehydrogenases/reductases that are involved in plant signal transduction regulated by various sterols [57]. In conclusion, the detection of unigenes that are involved in oleosin, caleosin and steroleosin biosynthesis will contribute to future functional studies and improvements in production levels by metabolic engineering of yellow horn.

### Unigenes Related to FA Desaturation

Many types of enzyme participate in fatty acid desaturation in plants, and can be divided into two types. One type catalyses the formation of monounsaturated fatty acids from saturated fatty acids in plastids (16∶0 to 16∶1, 18∶0 to 18∶1); these contain only a soluble enzyme, acyl-ACP desaturase (AAD, EC: 1.14.19.2) [58]. The other type is located on the membranes of the endoplasmic reticulum and chloroplast and introduces double-unsaturated bonds at specifically defined positions (Δ12, Δ15 or Δ6) in fatty acids that are esterified to a glycerol backbone [59], including Δ12(ω6)-Desaturase (FAD2 and FAD6, EC:1.14.19.-), which desaturates oleic acid (18∶1) to form linoleic acid (18∶2), Δ15(ω3)-Desaturase (FAD3, FAD7 and FAD8, EC:1.14.19.-), which further desaturates linoleic acid (18∶2) to form α-linolenic acid (18∶3), *etc*. In total, six, five, two, one, five and one unigenes that encode AAD, FAD2, FAD3, FAD6, FAD7 and FAD8 were found (Table 3). Oleic (18∶1) and linoleic acids (18∶2) are major constituents of yellow horn oil, according to a previous study, and ideal biodiesel should contain at least one double bond [19]; therefore, AAD, FAD2 and FAD6 are potential biotechnological targets for adjusting yellow horn oil composition.

### Unigenes Related to Catabolism Pathways for TAGs and FAs

The complete breakdown of TAGs can be divided into two steps [60]. First, TAGs are metabolised to free FAs; in other words, lipases catalyse the hydrolysis of ester bonds that link fatty acyl chains to the glycerol backbone. During this research, three unigenes that encode triacylglycerol lipase (TGL, EC: 3.1.1.3), which releases fatty acids and intermediate products (DAG or monoacylglycerol) from TAG or DAG, were identified in the transcriptome library (Table 4). In the second step, fatty acids are catabolised to acetyl-CoA, allowing them to be further broken down by oxidation or to follow other metabolic pathways, including re-esterification with glycerol, to form new acylglycerols [61]. Based on the KEGG pathway assignment, we identified 83 unigenes that code for enzymes related to fatty acid catabolism; three key enzymes were acyl-CoA oxidase (ACOX, EC: 1.3.3.6), enoyl-CoA hydratase/3-hydroxyacyl-CoA dehydrogenase (MFP2, EC: 4.2.1.17 1.1.1.35 1.1.1.211) and acetyl-CoA acyltransferase (ACAA, EC: 2.3.1.16), which were encoded by 11, 4 and 8 unigenes, respectively (Table 3). Acetyl-CoA generated through fatty acid catabolism is then used to produce energy for the cell via the citrate cycle or may participate in the synthesis of TAG.

TAG and FA catabolism proceeds in a direction opposite that of their synthesis. Therefore, identifying ways to suppress enzymes involved in TAG and FA catabolism may be another method of increasing the accumulation of lipids under conditions that do not affect plant growth. However, suppressing the expression of TGL increases TAG levels but results in severely stunted growth [62].

### Detection of Transcription Factors (TFs) Involved in Oil Synthesis

In previous studies, a set of TFs, including LEC1, LEC2, ABI3, FUS3 and WRI1 which play key roles in seed oil synthesis and deposition, were identified [63]–[65]. These TFs were reviewed by Fobert and made available online (http://lipidlibrary.aocs.org/plantbio/transfactors/index.htm).

To identify TFs that regulate seed oil synthesis, BLASTx was used to search against the AGRIS (*Arabidopsis* Gene Regulatory Information Server) database with an e-value cut off 10^−5^ [66]. The results showed that 3,341 unigenes were annotated with 905 independent coding sequences of *Arabidopsis* TFs belonging to 49 known TF families. However, none of the unigenes were annotated to the AtRKD family (Table S3). The largest number of unigenes (817) was annotated to the Trihelix family, followed by the C2H2 family (519 unigenes). TF genes in the PHD, CAMTA and JUMONJI families were identified in the yellow horn transcriptome.

Among the 3,347 unigenes, 25 encoding eight TFs involved in oil biosynthesis were detected (Table 5), including ABI3, L1L, ADOF1, EMF2, HSI2, HSI2-L1, AP2 and GL2. However, none of the unigenes showed homology to LEC1, LEC2, FUS3, WRI1, PKL, FIE or SWN.

**Table 5:** Putative transcription factors related to the oil biosynthesis in yellow horn.

TF Name	TF Family	Unigene number
ABI3 (Abscisic Acid Insensitive 3)	ABI3VP1	2
L1L (Leafy Cotyledon 1-Like)	CCAAT-HAP3	1
ADOF1 (Arabidopsis Dof Zinc Finger Protein 1)	C2C2-Dof	1
EMF2 (Embryonic Flower 2)	C2H2	5
HSI2 (High-Level Expression of Sugar-Inducible Gene 2)	ABI3VP1	5
HSI2-L1/HSL1 (HSI2-Like 1)	ABI3VP2	7
AP2 (APETALA2)	AP2-EREBP	1
GL2 (GLABRA2)	Homeobox	3

### Identification of Molecular Markers Located in Unigenes Related to Oil Biosynthesis and Metabolism

Simple sequence repeats (SSRs), single nucleotide polymorphisms (SNPs) and insertions and deletions (InDels) are valuable tools for genetic analysis because they are highly polymorphic within species. They can be used for molecular marker-assisted selection (MAS), which is a rapid approach to the development of new crop varieties (particularly in perennials and trees) that reduces the assessment time considerably [67], [68], association mapping to find genes related to good agronomic characteristics [69], and polymorphism analysis, among other techniques. Molecular markers located in genes will likely be related to the functions of those genes. Some markers that are located in coding regions related to oil synthesis have been developed and applied [70], [71].

In this study, a total of 6,707 SSRs distributed in 5,631 unigenes (4.61 Mb) were identified as potential molecular markers, of which 887 sequences contained more than one SSR. The most common SSRs were dinucleotide repeats, occurring at 3,598 loci (53.65%), followed by trinucleotide repeats (2,976, 44.37%). However, tetranucleotide, pentanucleotide and hexanucleotide repeats were found at lower frequencies, occurring only in 72 (1.15%), 17 (0.25%) and 44 (0.66%) SSRs, respectively (Table 6). Polymorphisms in these potential markers will the focus of future research.

**Table 6:** Number of SSRs in yellow horn.

Repeat motif	Repeat numbers							Number of SSRs	Percentage (%)
	**5**	**6**	**7**	**8**	**9**	**10**	**>10**		
Di-	–	1,052	709	535	395	257	650	3,598	53.65
Tri-	1,687	736	281	154	56	26	36	2,976	44.37
Tetra-	53	15	3	–	–	–	1	72	1.07
Penta-	15	2	–	–	–	–	–	17	0.25
Hexa-	32	8	1	–	1	2	–	44	0.66
Total	1,787	1,813	992	687	449	285	794	6,707	
Percentage (%)	26.64	27.03	14.82	10.27	6.74	4.25	10.24		

**Figure 5:**
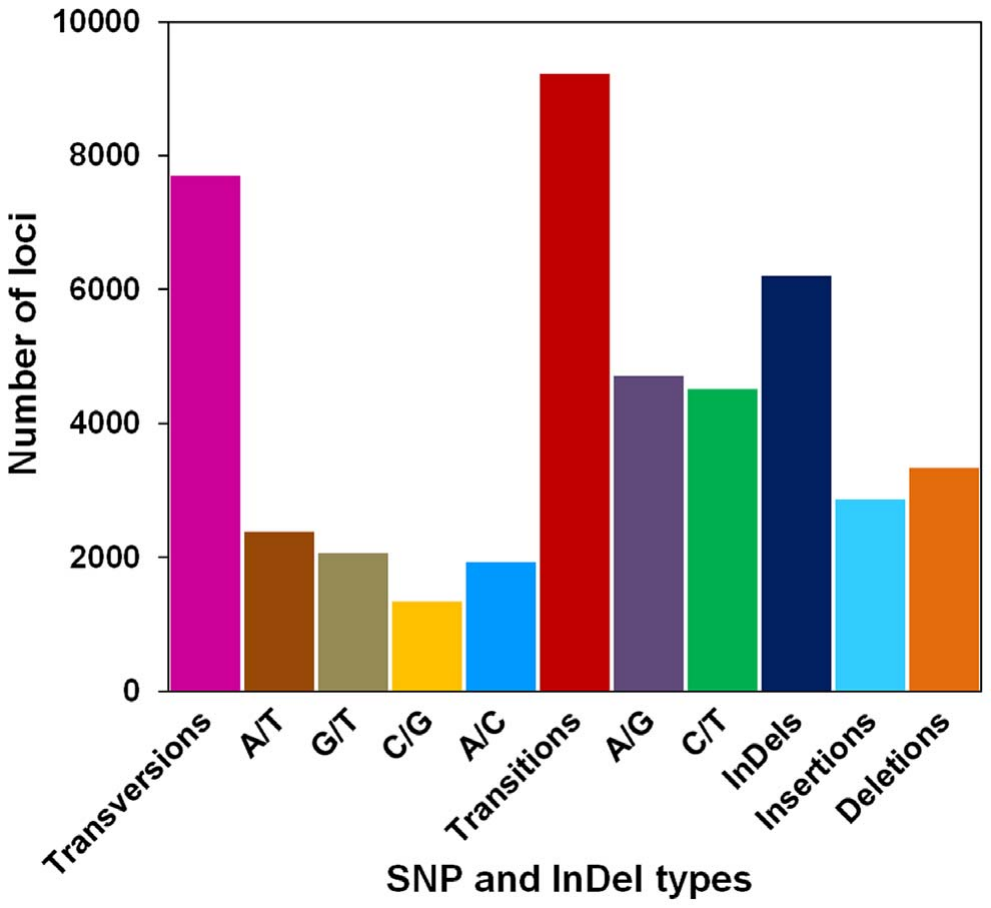
Distribution of putative single nucleotide polymorphisms (SNPs) and insertions and deletions (InDels) in the yellow horn transcriptome.

To increase the authenticity of SNP and InDel identification, we also filtered the results based on stricter multiple criteria, including read depth and allele frequency, compared with previous studies (see materials and methods). In total, 16,925 SNPs distributed across 4,401 different isotig groups that corresponded to 4,234 different unigenes had a total length of 6.10 Mb (Figure 5), resulting in an SNP occurrence rate of 0.003 per base and four SNPs per unigene. Transitions contained 9,225 (54.51%) SNPs and were primarily transversions (7,700 SNPs, 45.49%). The A/G and C/T transition genotypes had similar percentages, but among the four transversion genotypes, C/G transversions were less frequent than the other three (A/T, G/T, A/C). A total of 6,201 InDels were identified from 3,161 isotig groups that corresponded to 3,094 unigenes, with a total length of 4.41 Mb (Figure 5), which indicates there were two variations per unigene. Insertions (2,862, 46.15%) were slightly less common than deletions (3,339, 53.85%). The length of insertions ranged from 1 to 24 bp, and deletions were 1 to 22 bp in length.

We summed the SSRs, SNPs and InDels that were located in the 281 unigenes involved in the FA and TAG biosynthesis and metabolism pathways, which encoded oleosin, caleosin, steroleosin and TFs that regulated seed oil deposition. As a result, 26 SSRs, 194 SNPs and 60 InDels were found in 74 unigenes, which covered 31 enzymes or proteins and two TFs (ABI3 and HSI2-L1) (Table S4, Table S5 and Table S6). The remaining enzymes, such as MAT, EAR, FATA, ADH, ALDH, PCH, FAD6, PP and TGL4, and TFs, such as L1L, ADOF1, EMF2, HSI2, AP2 and GL2, contained no SSRs, SNPs or InDels.

Because we used larger samples from different plant regions and 1.25 runs to obtain the transcriptome, we found 29,833 (SSRs, SNPs and InDels) potential molecular markers in 9,720 unigenes (18.74%) with a total length of 10.37 Mb, for an average of 3.07 markers per unigene and spaces of 347.58 bp between markers. These markers will play significant roles not only in the production of genetically improved varieties of yellow horn with different oil compositions, increased oil yield and improved agronomic characteristics, but also for studying the evolution and origin of Xanthoceras, and even the Sapindaceae.

## Conclusions

In this study, we report the first comprehensive yellow horn sequencing effort using 454 GS FLX. Transcriptome analysis using four tissues (buds, leaves, flowers and developing seeds) of yellow horn found 51,867 unigenes (45 to 10,088 bp), which corresponded to 36.1 Mb and mean, N50 and median lengths of 696, 928 and 570 bp, respectively. These unigenes provide a strong basis for future genomic research to develop microarrays for gene expression assays and can serve as a reference transcriptome for future yellow horn RNA-seq experiments. In addition, 281 unigenes that code for key enzymes and TFs that are involved in reconstructed metabolic pathways for FA and TAG biosynthesis and metabolism were identified. Moreover, a large number of potential molecular markers (6,707 SSRs, 16,925 SNPs and 6,201 InDels) were predicted. Among them, 26 SSRs, 194 SNPs and 60 InDels were identified in 74 unigenes that are related to oil biosynthesis and metabolism. These findings will make a substantial contribution to efforts to improve crop characteristics and will accelerate the breeding of new yellow horn varieties.

## Materials and Methods

### Collection of Tissues for RNA Extraction

Yellow horn is widely distributed in China, so it has not been listed as an endangered or protected species. In this study, we used yellow horn buds, leaves (young and mature leaves), flowers, and developing seeds (10, 20, 30, 40, 50, 60, and 70 days after pollination) which were collected from 30 plants at the two locations. One is located in Xishan forest farm in Haidian, Beijing, China (E116°04′, N40°03′). This forest farm belongs to Beijing Forestry University and the yellow horn trees in this farm have been used for scientific research for several years. The other one is located in a small hill in Chengde city, Hebei province, China (E117°55′, N40°59′). The yellow horn trees which growing in this location were planted for scientific research by Dr. Ao Yan, a co-authors of this research. These samples were immediately frozen in liquid nitrogen and stored at −80°C. Because our research team is also engaged in the work of conservation and utilization of wild plant resources, we confirm that the field studies did not involve any endangered or protected species.

### cDNA Library Construction and 454 Sequencing

Total RNA was extracted separately from the buds, leaves, flowers and developing seeds using RNeasy Plant Mini Kits (Qiagen, Inc., Valencia, CA, USA) following the manufacturer’s protocol. Extracted RNA was qualified and quantified using a Nanodrop ND-1000 Spectrophotometer (Nanodrop Technologies, Wilmington, DE, USA) and all the samples showed a 260/280 nm ratio from 1.9 to 2.1. Poly(A)^+^ RNA was purified from total RNA by Oligotex mRNA Mini Kit (Qiagen, Inc., Valencia, CA, USA) following the manufacturer’s protocol. After that, equal quantities of total RNA from buds, leaves, flowers and seeds were mixed together. A total of 10 µg of total RNA was used for cDNA library construction. cDNA library construction and normalisation were performed using protocols described previously [72]. The resulting library was sequenced by means of one and a quarter 454 plate runs on a GS-FLX Titanium platform (Roche, USA).

### Analysis of 454 Transcriptome Sequencing Results

The raw reads were trimmed before assembly. First, adaptors and SMART primers that were used in the pyrosequencing reactions were cut using the SeqClean software. Then, we used the LUCY2 software [73] to remove low-quality regions and bases. Trimmed reads that were shorter than 45 bp were discarded and the remaining reads were assembled into isotigs and singlets using Newbler (version 2.6) [40]. Finally, after clustering the isotigs and singlets using CD-HIT (version 4.5.6) [74], the obtained unigenes were used in further analyses.

To understand their functions, the yellow horn unigenes were annotated using BLASTx alignment with an E-value cut-off of 10^−5^ against the following protein databases: NCBI non-redundant (NR), Swiss-Prot, Conserved Domain Database (CDD), Pfam protein families database (Pfam), UniProtKB/TrEMBL Protein Database (TrEMBL), Clusters of Orthologous Groups of proteins (COG), Gene Ontology (GO), Kyoto Encyclopaedia of Genes and Genomes (KEGG), and The Arabidopsis Information Resource (TAIR). GO functional classifications and KEGG pathway assignments were performed, as was described previously [75].

### Detection of SSRs, SNPs, InDels and TFs

To detect simple sequence repeats (SSRs) in the yellow horn transcriptome, the MISA (http://pgrc.ipk-gatersleben.de/misa/) software was used to identify all 2–6-bp motifs in the unigenes. The minimum repeat unit size was set at six for di-nucleotides and five for tri-, tetra-, penta-, and hexa-nucleotides.

The ssahaSNP software tool [76] was used to identify SNPs and InDels (1 to 100 bp in size) that had high coverage depths. To add the polymorphism and reliability of the SNPs and InDels, as in previous studies [26], [77], [78], we kept only SNPs and InDels that met the following strict quality criteria: (1) For SNP detection, we identified SNPs that had coverage depths of at least 20 and an alternate allele that was present at a minimum frequency of 20% in all isotigs that contained at least 20 reads. (2) For InDel detection, similar to the standard for SNPs, for insertions or deletions of one base, the coverage depth of the isotig and the InDel allele frequency were set at 20 and 20%, respectively. For insertions or deletions of two or more bases, the coverage depth of the isotigs and the InDel allele frequency were set at 10 and 10%, respectively.

To identify TFs, a BLAST search for all unigenes was conducted using AtTFDB (*Arabidopsis* transcription factor database) with shared identities >77%, as described previously [23].

### Data Deposition

The Roche 454 reads of yellow horn were deposited in the NCBI and can be accessed in the Short Read Archive (SRA) under accession number SRP026671.

## Supporting Information

Table S1 Pathway annotation of unignenes from yellow horn.
(XLS)

Tabel S2 Unigenes annotated as oleosin, caleosin and steroleosin.
(XLS)

Table S3 Putative transcription factors encoding unigenes in yellow horn.
(XLS)

Table S4 SSRs located in unigenes related to oil biosynthesis and metabolism.
(XLS)

Table S5 SNPs located in unigenes related to oil biosynthesis and metabolism.
(XLS)

Table S6 InDels located in unigenes related to oil biosynthesis and metabolism.
(XLS)
