# Correction: Transcriptome Analysis of Yellow Horn (*Xanthoceras sorbifolia* Bunge): A Potential Oil-Rich Seed Tree for Biodiesel in China

**DOI:** 10.1371/annotation/803f7e8c-0718-41b4-8fc2-cc0b5f776da9

**Published:** 2014-01-28

**Authors:** Yulin Liu, Zhedong Huang, Yan Ao, Wei Li, Zhixiang Zhang

The affiliation of the authors is incorrect. The correct affiliation is Beijing Forestry University, Beijing, China.

